# Universal and cultural factors shape body part vocabularies

**DOI:** 10.1038/s41598-024-61140-0

**Published:** 2024-05-07

**Authors:** Annika Tjuka, Robert Forkel, Johann-Mattis List

**Affiliations:** 1 Department of Linguistic and Cultural Evolution, Max Planck Institute for Evolutionary Anthropology, 04103 Leipzig, Germany; 2 Chair for Multilingual Computational Linguistics, University of Passau, 94032 Passau, Germany

**Keywords:** Semantics, Body parts, Cross-linguistic comparison, Language, Psychology

## Abstract

Every human has a body. Yet, languages differ in how they divide the body into parts to name them. While universal naming strategies exist, there is also variation in the vocabularies of body parts across languages. In this study, we investigate the similarities and differences in naming two separate body parts with one word, i.e., colexifications. We use a computational approach to create networks of body part vocabularies across languages. The analyses focus on body part networks in large language families, on perceptual features that lead to colexifications of body parts, and on a comparison of network structures in different semantic domains. Our results show that adjacent body parts are colexified frequently. However, preferences for perceptual features such as shape and function lead to variations in body part vocabularies. In addition, body part colexification networks are less varied across language families than networks in the semantic domains of emotion and colour. The study presents the first large-scale comparison of body part vocabularies in 1,028 language varieties and provides important insights into the variability of a universal human domain.

## Introduction

The languages of the world have different strategies for naming human body parts. English speakers have two words *foot* and *leg*, whereas Belhare speakers use one word, *laŋ*, to express the concepts foot and leg. Exploring the variation of body part vocabularies across languages has attracted the attention of researchers in linguistics, anthropology, and psychology over many years. Similar to the principles developed for the semantic domains of colour^1^, universal tendencies were established and contrasted with culturally specific variations^2–4^. The emergence of new methods in network analysis made it possible to conduct large-scale comparisons of vocabulary in specific semantic domains to examine universal and cultural structures^5,6^.

Variation in vocabularies is influenced by internal and external linguistic factors. Early comparative studies on the hierarchical structures of body part vocabularies across languages showed that they had generally five levels and never more than six^2^. In addition, general principles were established: (1) body parts such as head and arm are named in all languages, (2) leg and arm always receive distinct names, and (3) if a separate word exists for foot, then there will also be one for hand^7^. These cross-linguistic studies showed that visual discontinuity plays a role in the emergence of frequent patterns, for example, using the same word for the concepts hand and arm or for foot and leg^8^. The similarities in body part vocabularies were also used to decipher language relatedness since genealogically related languages overlap in strategies for naming body parts^9–11^. However, anthropological studies on body part vocabularies of diverse languages challenged the claims that the body part domain conforms to the same hierarchical principles across languages and that universal concepts such as body exist^3^. When considering linguistic diversity, the challenge is to identify the constraints that lead to the same outcome in different languages. Apart from visual features such as shape and contiguity, functional features, for example, that walking is performed by the foot and leg, are more important in some languages^8,12,13^. One possible explanation for why some languages colexify foot and leg or hand and arm is that their body part lexicon is based on the motor system used to perform actions, rather than the visual system^14^. The use of the same word for two body parts appears to be based on perception in general and the cultural significance of certain visual discontinuities in particular.

In this study, we investigate the structure of body part vocabularies across 1028 diverse languages. The overarching research question of the study is: What factors influence the variation of body part vocabularies across languages? The analyses are based on a large sample of lexical data and a computational approach to language comparison. We examine associations between body parts by analysing concepts that are expressed by the same word, i.e., colexifications^15^. We compute network comparisons across language families and examine body part colexifications in terms of their frequency and distribution. To investigate the internal structure of body part colexification networks, we compare network variation in the domain of body parts, colour, and emotion. Our study offers insights into the interplay between cognition and culture while demonstrating methodological advances in the computational analysis of cross-linguistic lexical data.

## Results

We conducted three different analyses to examine the structure of body part vocabularies across language varieties and language families. In total, 110 body part colexifications across 1028 language varieties were found. Table 1 includes the ten most frequent body part colexifications. The results reveal that concepts related to the limbs, i.e., parts of the arm and leg, are frequently colexified across different language families. In addition, body parts associated with the head are commonly referred to with the same word which is reflected in colexifications such as chin–jaw, mouth–lip, and eyebrow–eyelash. Part-of relations are expressed in colexifications such as breast–nipple and face–forehead.

**Table 1:** The 10 most frequent body part colexifications.

Concept A	Concept B	Families	Language varieties
foot	leg	57	322
hand	arm	37	255
chin	jaw	32	48
breast	nipple	29	45
finger	toe	28	104
breast	chest	19	47
mouth	lip	16	78
eyebrow	eyelash	14	45
finger	hand	14	18
face	forehead	12	14

The comparison of cross-linguistic patterns reveals that universal tendencies described in previous studies exist^2,7,10^. There is only one exception to the principle that if hand and foot are labelled, they are named by different words: Washo colexifies hand–foot. The general principle that a separate word for leg implies a separate word for arm^7^ is generally supported although there is one Nakh-Daghestanian language variety, Budukh, which colexifies leg–arm. The tendency of adjacent body parts to share the same name^9,10^ is supported by multiple body part colexifications and leads to cross-linguistically frequent patterns. While these overarching patterns reveal uniformity across the structure of body part vocabularies in diverse language varieties, many language family-specific patterns arise.

### Language family network comparison

**Figure 1:**
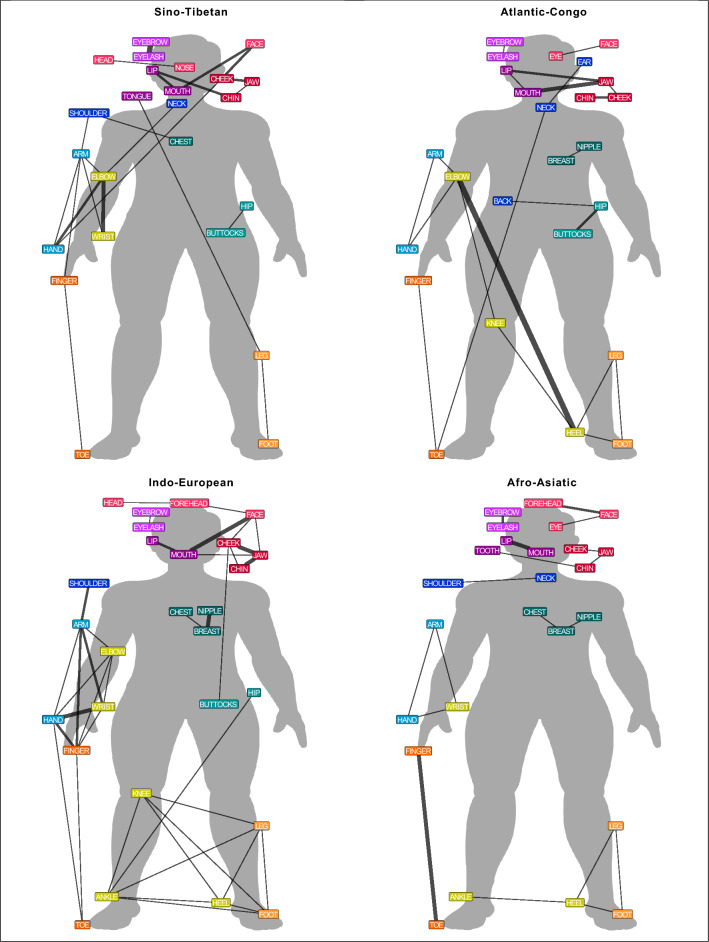
Language family colexification networks (1/2). The graph represents a weighted network in which the thickness of the edges indicates the frequency of a colexification across language varieties in a language family. The colour of the nodes shows the membership to the overall community.

**Figure 2:**
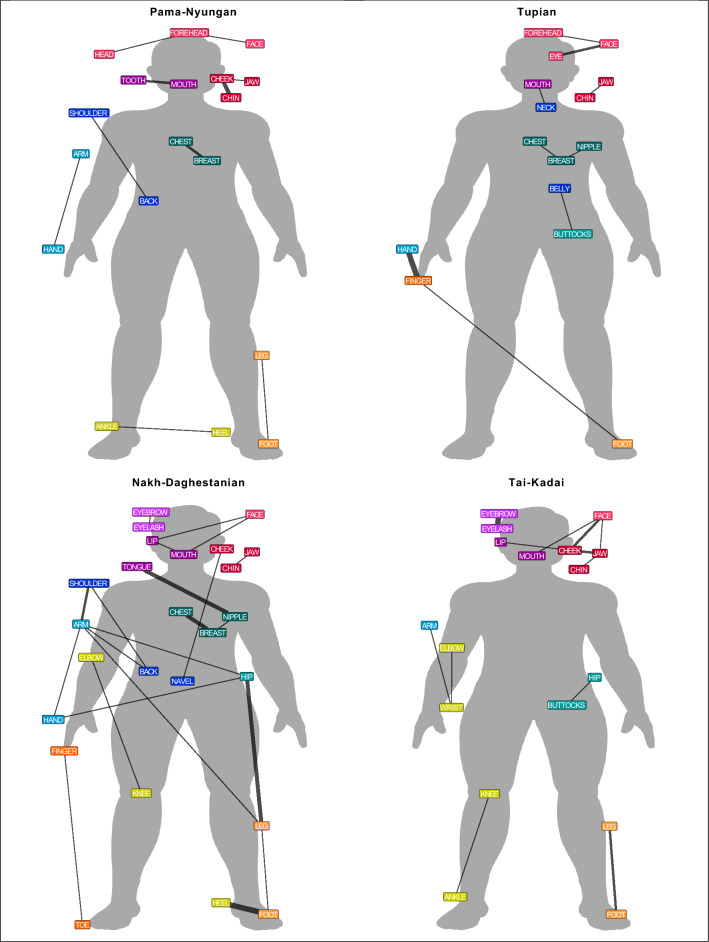
Language family colexification networks (2/2). The graph represents a weighted network in which the thickness of the edges indicates the frequency of a colexification across language varieties in a language family. The colour of the nodes shows the membership to the overall community.

Most colexifications between body part concepts occur in one or two language families. This shows that numerous body part colexifications are specific to a particular language family. Thus, we created language family networks for the eight language families with the highest number of language varieties in our sample to compare differences. The comparison of the language family networks shows striking variation in terms of the frequency and distribution of body part colexifications. Table 2 shows the number of body part colexifications in each language family. The comparison of the colexification frequencies demonstrates that a higher number of language varieties in a given language family does not lead to an increase in the number of body part colexifications. To compare the frequency and distribution of body part colexifications across the body, Figs. 1 and 2 illustrate the colexification networks for the eight language families. The colour of the nodes shows the membership to a community, with communities representing groups of nodes that have more connections among themselves than to other nodes in a network^16^.

**Table 2:** Number of body part colexifications across language families.

Family	Colexifications	Language varieties
Sino-Tibetan	22	151
Atlantic-Congo	22	117
Indo-European	38	57
Afro-Asiatic	18	61
Pama-Nyungan	10	61
Tupian	9	42
Nakh-Daghestanian	21	34
Tai-Kadai	12	28

The comparison of the network structures across eight language families reveals linguistic variation. While Sino-Tibetan and Indo-European language varieties have colexifications between different parts of the arm, only Indo-European language varieties show different colexifications of parts of the leg. In Atlantic-Congo language varieties, the colexification elbow–heel is frequent and it is specific to this language family. Afro-Asiatic, Pama-Nyungan, Tupian, and Tai-Kadai language varieties have primarily colexifications between adjacent body parts whereas Nakh-Dagestanian language varieties have multiple colexifications between non-adjacent body parts. Tupian is the only family in which no language variety shows a colexification between hand–arm and foot–leg.

The results of the descriptive comparison demonstrate that the structure of body part vocabularies varies across language families. Each language family has several body part colexifications that occur in 1-2 language varieties and many language family-specific body part colexifications exist. Often body part colexifications are confined to one area of the body and some language families tend to colexify different parts of a particular area of the body. While our approach provides an overview of the different patterns, studies on language subgroups^9^, genealogically related languages^17,18^, or individual languages^3,19,20^ offer a detailed comparison of systematic preferences within language families.

### Contiguity, function, and shape

**Figure 3:**
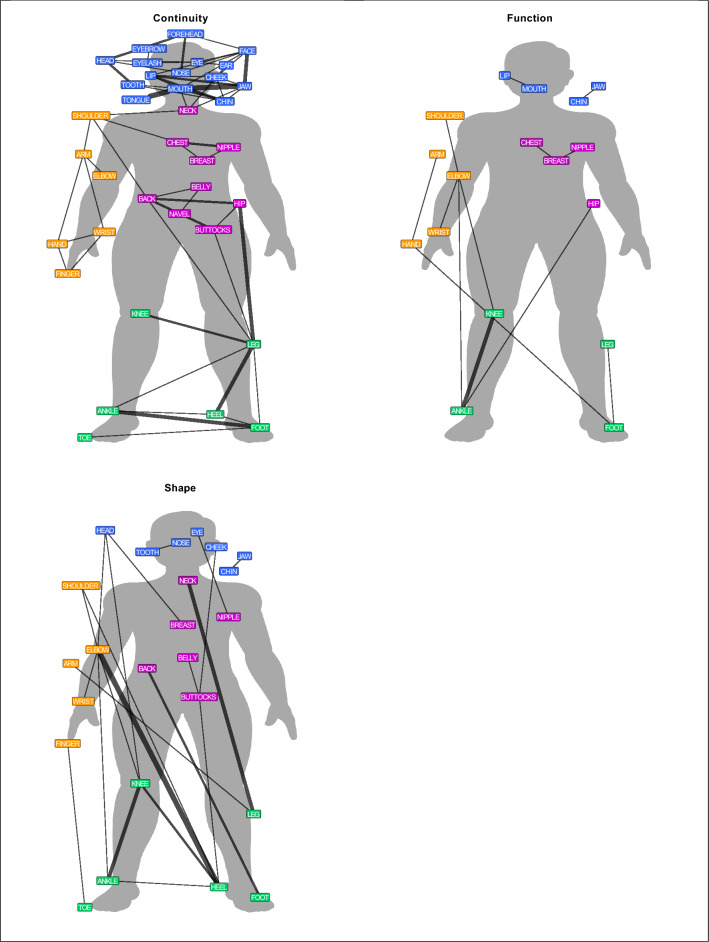
Colexification networks illustrating contiguity, function, and shape. The networks show colexifications based on contiguity (upper left), function (upper right), and shape (bottom left). The colours indicate the major parts of the body: head (blue), upper limb (orange), trunk (pink), and lower limb (green).

Each of the 110 body part colexifications was coded for three perceptual features: contiguity, function, and shape. Figure 3 shows the networks with the body part colexifications associated with a particular perceptual feature across 20 language families. The network based on body part colexifications associated with contiguity is the densest compared to the other two networks. This demonstrates that most cross-linguistic colexifications between body parts are based on a contiguous relation. While the networks of contiguity and function include cross-linguistically frequent body part colexifications, the majority of colexifications based on shape are language family-specific. For example, Manep, a language of the Nuclear Trans New Guinea family, colexifies head and knee with the word *kumu*.

**Figure 4:**
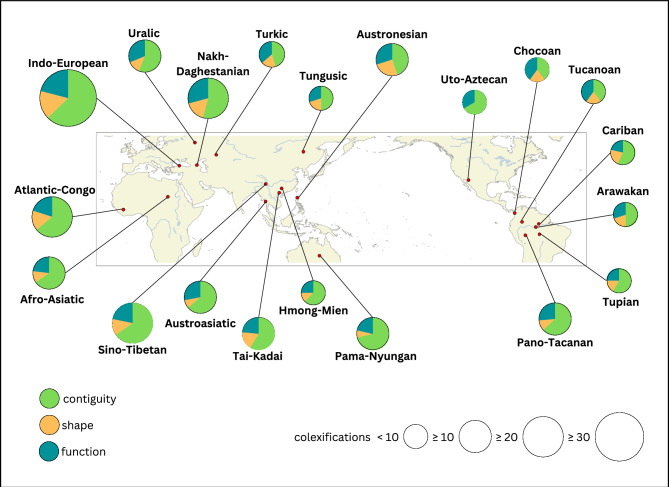
Distribution of the three perceptual features across language families. The perceptual features are contiguity, function, and shape. The size of the pie charts corresponds to the number of cross-linguistic colexifications present in the languages. The map illustrates the geo-coordinates for the origin of languages belonging to the same language family.

Based on the coding for contiguity, function, and shape, we determined the proportions of the categories for the 20 language families. Figure 4 illustrates the proportion of the three perceptual features across language families. The pie charts show the total number of colexifications associated with each perceptual feature in a given language family. The language families vary in their total number of colexifications which is illustrated by the size of the pie chart. For example, Indo-European has a total of 38 body part colexifications, whereas Uto-Aztecan only has four. The map shows the geographical origin of the language families to illustrate their spread across the globe.

The main result is that contiguity is prevalent in all language families. While Austronesian, Turkic, Chocoan, and Tucanoan languages have fewer body part colexifications associated with contiguity than all other language families, contiguity has still the highest proportion. There are slight cross-linguistic differences in the proportions of the body part colexifications associated with certain perceptual features. For example, Indo-European languages have an almost even number of body part colexifications associated with function and shape. Similar patterns are found in Atlantic-Congo and Tupian. In Arawakan and Tungusic languages, half of the body part colexifications are based on contiguity and the other half is split between shape and function. In contrast, there are no language families in which shape outweighs function. Most of the language families such as Uralic, Austroasiatic, Pama-Nyungan, or Tucanoan have more body part colexifications related to function over shape. Uto-Aztecan is the only language family that has no body part colexifications based on shape.

The geographical distribution of perceptual features shows interesting patterns. Contiguity is a cross-linguistically stable dimension that indicates universality. In contrast, the perceptual features of shape and function are culturally varied and languages employ different systems to structure their body part vocabularies. Multiple factors may lead to different preferences. In some regions, it is more likely to find languages that colexify body parts based on their function. These languages could be more likely to highlight actions systematically in their grammar by having a word order that places the verb at the beginning or by using a different system of verb agreement for transitive versus intransitive verbs. The perceptual feature of shape is frequent in only a few languages. However, some languages use this feature systematically for colexifications between body parts. One reason for this could be that these languages use shape markers to describe objects or have classifier systems based on shape features. The cultural variation and the interplay between lexicon and grammar could be further analysed with information about the grammatical structures of languages from the Grambank database^21^.

### Variation in body part, emotion, and colour networks

**Figure 5:**
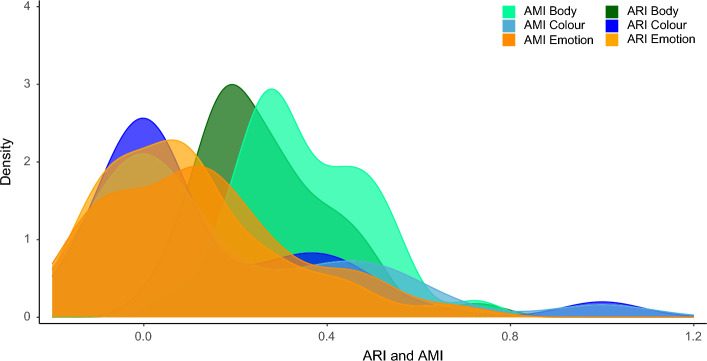
Distribution of pairwise language family ARI and AMI values for body, emotion, and colour networks with a 5-step random walk. The density plots show the distributions of ARI values for the domains of body part (green), emotion (orange), and colour (blue), and the distributions of AMI values for body part (dark green), emotion (dark orange), and colour (dark blue).

For comparing the variation in colexifications across three different semantic domains, we selected 20 language families with the highest number of languages in which colexifications of at least two out of three domains occurred. The comparison is a replication of the study by Jackson and colleagues^6^ which examined variation in colexifications in the domain of emotion and colour. We extended the list of concepts and created a new list with 21 colour concepts and 62 emotion concepts. To compare the differences in network structures, we derived adjusted rand index (ARI) and adjusted mutual information (AMI) values, illustrated in Fig. 5. The ARI values provide information on how similar the network clusters are compared to each other and give a measure for comparing whether two nodes remain in the same cluster, i.e., the rand index^22^. The AMI values also provide information on the similarities of clusters but they are more suited for networks with small clusters^23^. The resulting index lies between 0 and 1 corresponding to *completely random* and *completely identical*.

We performed an analysis with a 5-step random walk in line with the approach by Jackson et al.^6^ The analysis shows that the networks in the body part domain had a mean ARI of 0.3 (*sd* = 0.17). In comparison, the mean ARI of the emotion networks was 0.16 (*sd* = 0.29) and for colour *m* = 0.14 (*sd* = 0.26). The mean values of the AMI comparison across the three domains yielded similar results: body part *m* = 0.37 (*sd* = 0.16), emotion *m* = 0.18 (*sd* = 0.3), and colour *m* = 0.16 (*sd* = 0.28).

To test whether the variance in network clustering differed across domains, we performed Welch two sample *t*-tests with the mean ARI values. The results show that the body part networks vary significantly from the emotion networks with a higher variance in the emotion networks (*t* = 5.58, *p *< 0.001). The comparison with colour networks also shows a significant difference (*t* = 5.9, *p *< 0.001). The variance of clusters in the emotion and colour networks do not show a significant difference (*t* = 0.76, *p* = 0.45). The findings demonstrate that body part networks are more uniformly structured than emotion and colour networks. However, the finding that emotion networks varied significantly from colour networks was not replicated. The reason for the discrepancy could be the additional emotion and colour concepts that were added in the present analysis. They may have added more variation in the colour clusters.

**Figure 6:**
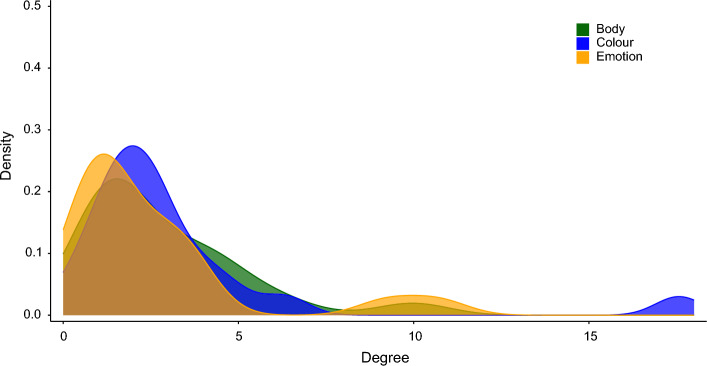
Distribution of language family weighted degrees for body, emotion, and colour networks. The density plot shows the frequency for the domains of body part (green), emotion (orange), and colour (blue).

Due to the low number of edges in the networks, we performed a third analysis to test the degree of edges in each language family. We ran 1000 trials selecting *n* nodes randomly in each trial, with *n* being the size of the selected nodes in the body, colour, and emotion networks. From these random selections, weighted degrees for selected nodes in the network per language family for each semantic domain were computed. Figure 6 illustrates the distribution of the language family weighted degrees in proportion to the number of language varieties for the domains of body part, emotion, and colour.

The plot shows that the distribution of the weighted degrees differs slightly across the three domains. The mean weighted degree in the body part domain was 2.87 (*sd* = 2.3). In the emotion domain, the mean was 2.6 (*sd* = 2.75) and in the colour domain, the mean was 3.21 (*sd* = 3.69). This result demonstrates that across the 20 language families in our sample, colour colexifications have the highest degree of edge connections, followed by body part and emotion. The comparison of the weighted degrees based on a Welch two sample *t*-test across domains showed no significant difference between the three domains. The findings indicate that the degree of edge connections in body part, emotion, and colour colexification networks is similar.

## Discussion

Body part vocabularies vary across languages. However, within the diversity, general tendencies arise. Body parts that are adjacent to one another are more frequently colexified. At the same time, linguistic diversity arises due to preferences for colexifications based on a perception of shape or function. Our study provided a first analysis of colexifications in body part vocabularies across 1028 languages. The results showed that uniform structures arise across language families indicating that body part vocabularies are not random. In addition, we demonstrated that body part colexification networks differ significantly from the domains of emotion and colour in that body part colexification networks are less varied across language families.

Apart from theoretical implications, the study demonstrates three important methodological improvements to the study of colexification networks. The first improvement is the use of Lexibank^24^ as the basis for the data in combination with the workflows in CLICS^3^^25^ which makes our approach more flexible for future applications to other semantic domains. We included datasets with large coverage and from different geographical areas. In a subsequent study, the data need to be optimised to reach a genealogical and geographically balanced sample. We did not restrict our sample in the present study because it is the first large-scale study on body part colexifications so we aimed to get a broad perspective on the emerging patterns. The second methodological improvement is the inclusion of a cognate detection method to account for language relatedness^26^. Although preliminary tests did not detect noteworthy differences in the resulting colexification networks when different thresholds of cognates were considered, the method needs further testing and will become important in studies on individual language families. The third methodological improvement is the replication of the comparison of semantic domains^6^. By implementing the analysis in Python code, the underlying analysis is now more transparent and parts can be conveniently adapted. In addition, we compared weighted degrees across language families to examine the structure of colexification networks. This method is particularly important given the sparseness of the connections found in the three semantic domains and it allows us to bridge gaps in the data.

Future studies can use our workflows to add more language varieties or compare other semantic domains. Our approach was exploratory to some extent and a more balanced sample is required for further research. Although we included as many concepts as possible, the coverage of concepts is skewed across the world’s languages and further data collection is necessary. Our study provides the first large-scale analysis of body part vocabularies and offers insights into the structure of body part vocabularies in diverse languages which can lead to more robust interpretations of colexifications in different semantic domains.

## Methods

### Language sample

**Figure 7:**
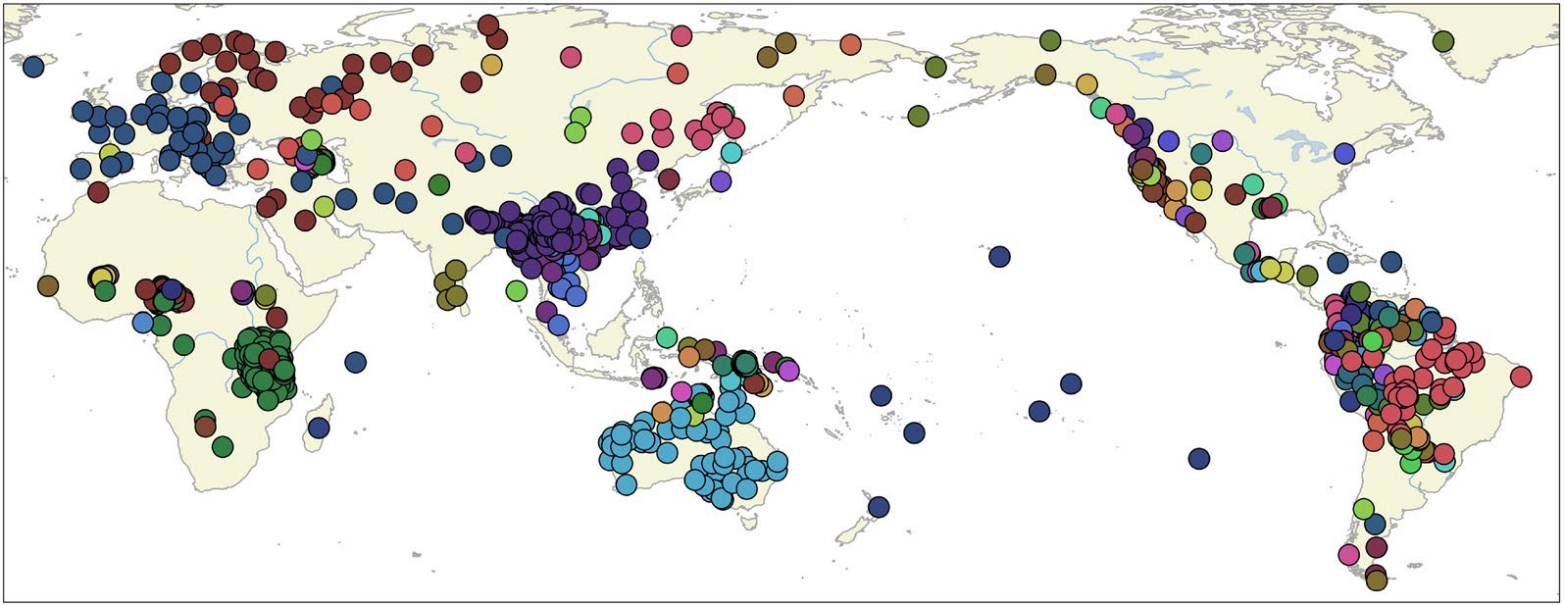
Distribution of language varieties in the sample. The colour indicates membership to a language family. The classification and coordinates are taken from Glottolog Version 4.7^27^, https://glottolog.org.

The study is based on a sample of 1028 language varieties from different geographic regions and 20 language families, see Fig. 7. Table 3 presents the 20 language families with the number of language varieties in the sample. The largest language families are Sino-Tibetan (151 language varieties), Atlantic-Congo (117 language varieties), and Pama-Nyungan (61 language varieties). We incorporated language families with a large number of language varieties to have sufficient coverage of body part, emotion, and colour concepts.

**Table 3:** Number of language varieties across language families.

Rank	Family	Language varieties
1	Sino-Tibetan	151
2	Atlantic-Congo	117
3	Pama-Nyungan	61
4	Afro-Asiatic	61
5	Indo-European	57
6	Tupian	42
7	Nakh-Daghestanian	34
8	Tai-Kadai	28
9	Uralic	26
10	Hmong-Mien	24
11	Austroasiatic	21
12	Tucanoan	18
13	Arawakan	15
14	Tungusic	13
15	Turkic	11
16	Uto-Aztecan	10
17	Austronesian	10
18	Pano-Tacanan	10
19	Cariban	9
20	Chocoan	6

### Concept selection

The body part concepts for the study were selected from the semantic field ‘The body’ in Concepticon Version 2.5^28^. A description of the selection process and the list are provided in blog posts^29,30^. We excluded body part concepts for which no corresponding word was available in the datasets and less than five words in at least ten language families were elicited. This process led to a selection of 36 body part concepts (Table 4). The lexical data were taken from 51 datasets consisting of word lists with at least 250 concepts curated in Lexibank^24^. The datasets are listed in the Supplementary Information.

**Table 4:** Body part concepts.

ID	Concept		ID (cont.)	Concept (cont.)
1402	breast		1303	finger
834	buttocks		123	forehead
1173	eyebrow		1277	hand
1301	foot		1256	head
980	heel		1745	hip
1371	knee		798	jaw
803	ankle		1297	leg
1673	arm		478	lip
1291	back		674	mouth
1251	belly		1838	navel
1730	cheek		1333	neck
1592	chest		796	nipple
1510	chin		1221	nose
1247	ear		1482	shoulder
981	elbow		1389	toe
1248	eye		1205	tongue
1540	eyelash		1380	tooth
1560	face		799	wrist

The concepts are based on Concepticon Version 2.5^28^

The study includes an additional comparison with concepts from other semantic domains, i.e., emotion and colour. Thus, we collected the emotion and colour concepts used by Jackson and colleagues^6^ and extended the list with concepts available in Concepticon Version 2.5^30,31^. The final list included 22 colour concepts and 62 emotion concepts (see Supplementary Information).

### Workflows

#### Colexification networks

The workflow of identifying colexification networks is based on the workflows that are the foundation for creating the network in CLICS^3^^25^. Apart from computing the frequencies of cross-linguistic colexifications of a given set of concepts, the computer-assisted approach using the CLICS algorithm creates a weighted network of colexifications^32,33^. The graphs were produced with the Python package NetworkX^34^. To identify communities within these networks, we employed the Infomap algorithm^35^ integrated into the Python package igraph^36^. The Supplementary Information provides additional details on the methods.

#### Cognate detection

To account for language relatedness in the emergence of colexifications, we established a new method that detects cognates in genealogically related languages to identify whether a colexification was transmitted from a shared ancestor language. We employed an automated approach to generating phonetic transcriptions based on the cross-linguistic transcription systems (CLTS) reference catalogue^37^, https://clts.clld.org. These phonetic transcriptions are now incorporated in Lexibank^24^ and allow a comparison of sounds rather than symbols^38^.The method computes all colexifications inside the same family and then automatically clusters all word forms that colexify the same concepts across different language varieties into cognate sets. We used state-of-the-art methods for automated cognate detection^26^, as implemented in LingPy Version 2.6.13^39^ (https://lingpy.org).

While previous approaches list all language varieties for which a colexification inside a given family could be detected^6^, our revised approach counts only the number of distinct cognate sets. As a result, we capture cases where a colexification evolved only once in the past and was then transmitted to all neighbouring languages in a sample. For example, in the Austronesian language family, most language varieties use the word forms *lima* or *nima* for the colexification hand-five. Since the cognate detection method detects that *lima* and *nima* are cognate, it assigns both words to the same cluster and thus guarantees that we count the colexification only once, instead of counting it multiple times. Preliminary tests show no striking differences in colexification networks of body part concepts with different thresholds for cognate detection, but the method needs further examination in subsequent studies.

#### Language family origin detection

The origins of language families, i.e., homelands, were computed using an algorithm implemented in the homelands module provided by the Python package pyglottolog (https://pypi.org/project/pyglottolog/3.11.0). Geographic point locations for Glottolog subgroups (https://glottolog.org) are determined recursively as the nearest point on land to the intersection centroid of the coordinate set of immediate daughter languages or subgroups (https://pyglottolog.readthedocs.io/en/latest/homelands.html#module-pyglottolog.homelands). This method is used for visualisation purposes only, i.e., to illustrate language families on a map, not as the basis for quantitative analysis of the origins of language families.

#### Perceptual features

The perceptual features *contiguity*, *shape*, and *function* offer important insights into the structure of body vocabularies across languages^7,8,14^. We, therefore, coded each body part colexification for *presence*/*absence* (1/0) of a perceptual feature. Since some of the body part colexifications can be interpreted in terms of different features, we allowed for multiple coding of presence. The colexification hand-arm, for instance, was coded as follows: contiguity 1, shape 0, and function 1. In comparison, the colexification head-knee was coded as contiguity 0, shape 1, and function 0. The full list of coding is given in the Supplementary Information.

### Supplementary Information

Supplementary Information.

## Data Availability

The Supplementary Information provides an overview of the datasets, concepts, and coding. The data and scripts for the analysis of this study are accessible on GitHub (https://github.com/clics/clicsbp/releases/tag/v1.0) and stored on Zenodo (https://doi.org/10.5281/zenodo.10955934).

## References

[1] Berlin B, Kay P (1969). Basic Color Terms: Their Universality and Evolution.

[2] Brown CH (1976). General principles of human anatomical partonomy and speculations on the growth of partonomic nomenclature. Am. Ethnol..

[3] Majid A, Enfield N J, van Staden M (2006). Parts of the body: Cross-linguistic categorisation (Special Issue). Lang. Sci..

[4] Wierzbicka A (2007). Bodies and their parts: An NSM approach to semantic typology. Lang. Sci..

[5] Youn H (2016). On the universal structure of human lexical semantics. Proc. Natl. Acad. Sci. Biol. Sci..

[6] Jackson JC (2019). Emotion semantics show both cultural variation and universal structure. Science.

[7] Andersen ES, Greenberg JH (1978). Lexical universals of body-part terminology. Universals of Human Language: Word Structure.

[8] Majid A, Malt BC, Wolff P (2010). Words for parts of the body. Words and the Mind: How Words Capture Human Experience.

[9] Matisoff JA (1978). Variational Semantics in Tibeto-Burman: The “Organic” Approach to Linguistic Comparison.

[10] Wilkins DP, Durie M, Ross M (1996). Natural tendencies of semantic change and the search for cognates. The Comparative Method Reviewed: Regularity and Irregularity in Language Change.

[11] Majid A, Jordan F, Dunn M (2015). Semantic systems in closely related languages. Lang. Sci..

[12] Tversky B (1989). Parts, partonomies, and taxonomies. Dev. Psychol..

[13] Morrison JB, Tversky B (2005). Bodies and their parts. Mem. Cogn..

[14] Majid A, van Staden M (2015). Can nomenclature for the body be explained by embodiment theories?. Top. Cogn. Sci..

[15] François A, Vanhove M (2008). Semantic maps and the typology of colexification: Intertwining polysemous networks across languages. From Polysemy to Semantic Change: Towards a Typology of Lexical Semantic Associations.

[16] Newman M (2006). Modularity and community structure in networks. Proc. Natl. Acad. Sci. U.S.A..

[17] McClure EF (1975). Ethno-anatomy: The structure of the domain. Anthropol. Linguist..

[18] Huisman JLA, van Hout R, Majid A (2021). Patterns of semantic variation differ across body parts: Evidence from the Japonic languages. Cogn. Linguist..

[19] Stark LR (1969). The lexical structure of Quechua body parts. Anthropol. Linguist..

[20] Palmer GB, Nicodemus L (1985). Coeur d’Alene exceptions to proposed universals of anatomical nomenclature. Am. Ethnol..

[21] Skirgård H (2023). Grambank reveals the importance of genealogical constraints on linguistic diversity and highlights the impact of language loss. Sci. Adv..

[22] Newman M (2018). Networks: An Introduction.

[23] Romano S, Vinh NX, Bailey J, Verspoor K (2016). Adjusting for chance clustering comparison measures. J. Mach. Learn. Res..

[24] List J-M (2022). Lexibank, a public repository of standardized wordlists with computed phonological and lexical features. Sci. Data.

[25] Rzymski C (2020). The database of cross-linguistic colexifications, reproducible analysis of cross-linguistic polysemies. Sci. Data.

[26] List J-M, Greenhill SJ, Gray RD (2017). The potential of automatic word comparison for historical linguistics. PLOS ONE.

[27] Hammarström H, Forkel R, Haspelmath M, Bank S (2022). Glottolog (Version 4.7).

[28] List J-M (2021). Concepticon. A Resource for the Linking of Concept Lists (Version 2.5.0).

[29] Tjuka A (2020). A list of 171 body part concepts. Comput. Assist. Lang. Comp. Pract..

[30] Tjuka A (2021). A list of color, emotion, and human body part concepts. Comput. Assist. Lang. Comp. Pract..

[31] Tjuka A (2022). Extending the list of color, emotion, and human body part concepts. Comput. Assist. Lang. Comp. Pract..

[32] Mayer, T., List, J.-M., Terhalle, A. & Urban, M. An interactive visualization of crosslinguistic colexification patterns. In *Proceedings of the LREC Workshop ’VisLR: Visualization as Added Value in the Development, Use and Evaluation of Language Resources’* (eds Hautli-Janisz, A. *et al.*) 1–8 (European Language Resources Association, Reykjavik, Iceland, 2014).

[33] List, J.-M. Towards a History of Concept List Compilation in Historical Linguistics. *History and Philosophy of the Language Sciences* (2018).

[34] Hagberg, A. A., Schult, D. A. & Swart, P. J. Exploring Network Structure, Dynamics, and Function Using NetworkX. In Varoquaux, G., Vaught, T. & Millman, J. (eds.) *Proceedings of the 7th Python in Science Conference (SciPy2008)*, 11–15 (Pasadena, United States, 2008).

[35] Rosvall M, Bergstrom CT (2008). Maps of random walks on complex networks reveal community structure. Proc. Natl. Acad. Sci..

[36] Csárdi G, Nepusz T (2006). The igraph software package for complex network research. Inter J. Complex Syst..

[37] List J-M, Anderson C, Tresoldi T, Forkel R (2021). Cross-Linguistic Transcription Systems (Version 2.2.0).

[38] List J-M (2023). Inference of partial colexifications from multilingual wordlists. Front. Psychol..

[39] List J-M, Forkel R (2023). LingPy. A Python Library for Quantitative Tasks in Historical Linguistics [Software Library, Version 2.6.13].

