# Supplementary material for: Universal and cultural factors shape body part vocabularies
Source: Sci Rep. 2024 May 7;14:10486. doi: 10.1038/s41598-024-61140-0 (PMC11076558; doi:10.1038/s41598-024-61140-0)
Supplement: Supplementary file 1 — Supplementary Information. [file 41598_2024_61140_MOESM1_ESM.pdf]

Supplementary Information
for 'Universal and cultural factors shape body part vocabularies'

Annika Tjuka[1,*], Robert Forkel[1], and Johann-Mattis List[1,2]

[1]Department of Linguistic and Cultural Evolution, Max Planck Institute for Evolutionary Anthropology, Leipzig, 04103, Germany
[2]Chair for Multilingual Computational Linguistics, University of Passau, Passau, 94032, Germany
[*]annika_tjuka@eva.mpg.de

ABSTRACT

Every human has a body. Yet, languages differ in how they divide the body into parts to name them. While universal naming strategies exist, there is also variation in the vocabularies of body parts across languages. In this study, we investigate the similarities and differences in naming two separate body parts with the same word, i.e., colexifications. We use a computational approach to create networks of body part vocabularies across languages. The analyses focus on body part networks in large language families, on perceptual features that lead to colexifications of body parts, and on a comparison of network structures in different semantic domains. Our results show that adjacent body parts are colexified more frequently. However, preferences for perceptual features such as shape and function lead to variations in body part vocabularies. In addition, body part colexification networks are less varied across language families than networks in the semantic domains of emotion and colour. The study presents the first large-scale comparison of body part vocabularies in 1,028 language varieties and provides important insights into the variability of a universal human domain.

1 Extended method description

Supplementary Table 1. Extended method description. The data and scripts for the analysis of this study are accessible on GitHub (https://github.com/clics/clicsbp/releases/tag/v1.0) and stored on Zenodo (https://doi.org/10.5281/zenodo.10955934).

Method	Files in `clicsbpcommands` Folder	Description
Colexification networks	`colexifications.py`, `colexify_all_data.py`	A colexification occurs when the same word form is used for two different concepts. For example, the word form *laŋ* in Belhare is used for the concepts FOOT and LEG. By identifying the word forms that colexify body part concepts, a network with connections between the concepts that share the same word form is created. Since some concepts are colexified more frequently than others across languages, we build a weighted network in which the weights represent the frequency of colexifications between concepts across languages or language families.[1–3] The colexification networks are built out of the lists of concepts and the word forms stored in the datasets in the `clicsbp/etc` folder. The output is a graph in GML file format and a matrix of the colexifications in TSV file format.

Community detection	`colexifications.py`, `colexify_all_data.py`	A community in a network is a group of nodes that are densely connected. Since not all nodes in sparse networks show connections to all other nodes in the network, it is difficult to assess the relation between unconnected nodes. The Infomap algorithm[4] identifies the communities based on random walks that start at a node in the network and then move to a random neighboring node.[5] By defining a specific number of steps that can be taken, the walk moves along the edges of connecting nodes until the number is reached and the connected nodes are identified as a community. If multiple nodes are densely connected, the random walk is more likely to stay in this group of nodes. The random walks provide a way to achieve direct similarity scores for all node pairs in a network. The core idea is to simulate a random walk through the network and determining how frequently two nodes meet. Our reimplementation of Jackson et al. (2019)[6] can be found in the Python script `colexifications.py` (lines 176 to 203) in the folder `clicsbp/clicsbpcommands`.
Cognate detection	`colexifications.py`	The script uses the Sound-Class-Based Phonetic Alignment (SCA) method for automated cognate detection[7] with a threshold of 0.45 (function `weight_by_cognacy` in the Python script) and Infomap[4] as implemented in the `igraph`[8] package as the basic method for cognate clustering. The method proceeds by first assembling all individual word forms that are found to support a given colexification and compares these word forms using the Sound-Class-Based Phonetic Alignment algorithm[7,9] to obtain individual distances for each word form. These distances are used to construct a graph in which all word forms beyond the distance of 0.45 (which was empirically determined in previous studies)[7] are interlinked. The graph is analysed with the Infomap algorithm to detect communities, with communities reflecting individual cognate sets.
Network structure comparison	`ari.py`	The pairwise comparisons of language family network community structures were performed using Adjusted Rand Index (ARI) and Adjusted Mutual Information (AMI) values. The similarity between network clusters is determined by comparing whether two nodes remain in the same cluster, i.e., the Rand Index.[5] While the ARI values are suited for networks with large clusters, the AMI values are used for networks with small clusters.[10] The index is between 0 and 1 corresponding to *completely random* and *completely identical*. Thus, the lower the value the more variation between network clusters.
Degree comparison	`degrees.py`	The colexification networks have weighted degrees based on the frequency of a given colexification across languages or language families. By selecting n nodes randomly for the networks of the three semantic domains body, colour, and emotion, we created random selections within 1,000 trials. The weighted degrees for the node selection in the network per language family were then computed for each semantic domain.

2 Lexibank datasets

Supplementary Table 2. List of the Lexibank datasets included in the analysis of body-object colexifications. All Lexibank datasets are stored on GitHub: github.com/lexibank.

Lexibank ID	Collection	Citation
aaleykusunda	LexiCore	11
abrahammonpa	ClicsCore	12
allenbai	ClicsCore	13
bantubvd	ClicsCore	14
beidasinitic	ClicsCore	15
bodtkhobwa	ClicsCore	16
bowernpny	ClicsCore	17
castrosui	ClicsCore	18
castroyi	ClicsCore	19
castrozhuang	ClicsCore	20
chenhmongmien	ClicsCore	21
chindialectsurvey	ClicsCore	22
clarkkimmun	ClicsCore	23
halenepal	ClicsCore	24
hantganbangime	ClicsCore	25
hubercolumbian	ClicsCore	26
hsiuhmongmien	ClicsCore	27
huntergatherer	ClicsCore	28
johanssonsoundsymbolic	ClicsCore	29
kraftchadic	ClicsCore	30
lindseyende	ClicsCore	31
listsamplesize	ClicsCore	32
luangthongkumkaren	ClicsCore	33
mannburmish	ClicsCore	34
marrisonnaga	ClicsCore	35
mitterhoferbena	ClicsCore	36
naganorgyalrongic	ClicsCore	37
northeuralex	ClicsCore	38
polyglottaafricana	ClicsCore	39
robinsonap	ClicsCore	40
sagartst	ClicsCore	41
simsrma	ClicsCore	42
sohartmannchin	ClicsCore	43
suntb	ClicsCore	44
tls	ClicsCore	45
transnewguineaorg	ClicsCore	46
tuled	ClicsCore	47
oskolskayatungusic	ClicsCore	48
visserkalamang	ClicsCore	49
wangbai	ClicsCore	50
wold	ClicsCore	51
yanglalo	ClicsCore	52
yangyi	ClicsCore	53
yuchinese	ClicsCore	54
zgraggenmadang	ClicsCore	55
othanieljen	ClicsCore	56
lamanisoic	ClicsCore	57
savelyevturkic	ClicsCore	58

oskolskayatungusic	ClicsCore	[48]
idssegmented	ClicsCore	[59]
zhivlovobugrian	ClicsCore	[60]
zhoubizic	ClicsCore	[61]

3 Body part concepts

Supplementary Table 3. **Body part concepts taken from Concepticon Version 2.5.0**[62].

ID	Concept	Domain	Broader Concept	Broader ID
153	GREY	color		
156	RED	color		
163	BLACK	color	BLACK OR DARK	2633
837	BLUE	color	BLUE OR GREEN	2382
1335	WHITE	color		
1424	YELLOW	color		
1425	GREEN	color	BLUE OR GREEN // GREEN OR UNRIPE	2382 // 2300
2409	BROWN	color		
2452	PURPLE	color		
3045	COPPER (COLOR)	color		
3328	ORANGE (COLOR)	color		
3446	PINK	color		
3604	LIGHT BLUE	color		
3605	LIGHT GREEN	color		
3706	DARK YELLOW	color		
3707	BEIGE	color		
3750	KHAKI	color		
3751	BRIGHT YELLOW	color		
3752	LILAC	color		
3753	MAGENTA	color		
3795	DARK GREEN	color		
3914	TURQUOISE	color		
40	ENVY	emotion		
82	ANGER	emotion		
781	FEAR (FRIGHT)	emotion		
1000	ANXIETY	emotion		
1141	PITY	emotion		
1668	GRIEF	emotion		
1776	SHAME	emotion		
1783	PAIN	emotion		
1912	RESPECT (NOUN)	emotion		
2016	JEALOUSY	emotion		
2465	DISGUST	emotion		
2524	JOY	emotion		
2553	MISERY	emotion		
2941	HAPPINESS	emotion		
2960	LONGING (WISH)	emotion		
3049	COURAGE	emotion		
3211	HOPE (FAITH)	emotion		
3468	KINDNESS	emotion		
3673	MOURNING	emotion		
3749	HATE (LOATHING)	emotion		
3844	CONTEMPT	emotion		
3845	DISAPPOINTMENT	emotion		
3846	EXCITEMENT	emotion		
3847	INTEREST (FEELING)	emotion		
3848	SURPRISE (FEELING)	emotion		
3849	TRIUMPH	emotion		
3850	AMUSEMENT	emotion		
3851	AWE	emotion		
3853	CONFUSION	emotion		

3854	SADNESS	emotion		
3877	DEPRESSION	emotion		
3915	PRIDE	emotion		
3916	PLEASURE	emotion		
3834	LOVE (AFFECTION)	emotion		
1552	GLOOMY	emotion		
1495	HAPPY	emotion		
1607	HATE	emotion		
1092	HOPE	emotion		
923	LOVE	emotion		
174	PROUD	emotion		
1875	REGRET	emotion		
699	SAD	emotion		
1572	SURPRISED	emotion		
150	WORRY	emotion		
1976	MERRY	emotion		
2117	DESIRE	emotion		
1035	GOOD	emotion		
1117	LIKE	emotion		
1292	BAD	emotion	BAD OR EVIL	3764
1784	WANT	emotion		
1826	ANGRY	emotion		
2716	BLAME (SOMEBODY)	emotion		
3299	KIND	emotion		
1308	DOUBT	emotion		
3762	SAFE	emotion		
1894	ASHAMED	emotion		
3885	IMPATIENT	emotion		
1900	ASTONISHED	emotion		
2821	JEALOUS	emotion		
3033	SCARED	emotion		
3884	PATIENT	emotion		
1955	GRIEVE	emotion		
1402	BREAST	human body part	BREAST OR MILK	2129
834	BUTTOCKS	human body part		
1173	EYEBROW	human body part		
1301	FOOT	human body part	FOOT OR LEG	2098
980	HEEL	human body part		
1371	KNEE	human body part		
803	ANKLE	human body part		
1673	ARM	human body part	ARM OR HAND	2121
1291	BACK	human body part		
1251	BELLY	human body part	BELLY OR STOMACH	2114
1730	CHEEK	human body part		
1592	CHEST	human body part		
1510	CHIN	human body part		
1247	EAR	human body part	EAR OR HEAR	2126
981	ELBOW	human body part		
1248	EYE	human body part		
1540	EYELASH	human body part		
1560	FACE	human body part		
1303	FINGER	human body part	FINGER OR TOE	2120
123	FOREHEAD	human body part	HAIR OR FORE-HEAD	2107
1277	HAND	human body part	ARM OR HAND	2121
1256	HEAD	human body part		
1745	HIP	human body part		
798	JAW	human body part		
1297	LEG	human body part	FOOT OR LEG	2098
478	LIP	human body part		
674	MOUTH	human body part		
1838	NAVEL	human body part		
1333	NECK	human body part		
796	NIPPLE	human body part		
1221	NOSE	human body part		
1482	SHOULDER	human body part		

1389	TOE	human body part	FINGER OR TOE	2120
1205	TONGUE	human body part		
1380	TOOTH	human body part		
799	WRIST	human body part		

Supplementary Table 4. Body part colexifications sorted by their frequency across language families and their coding for continuity, shape, and function.

Concept A	Concept B	Languages	Families	Continuity	Shape	Function
FOOT	LEG	326	55	1	0	1
HAND	ARM	256	36	1	0	1
CHIN	JAW	47	32	1	1	1
BREAST	NIPPLE	48	30	1	0	1
FINGER	TOE	104	29	0	1	0
BREAST	CHEST	56	19	1	0	1
MOUTH	LIP	79	16	1	0	1
EYEBROW	EYELASH	47	14	1	0	0
FINGER	HAND	19	14	1	0	0
FACE	FOREHEAD	15	12	1	0	0
CHEEK	FACE	16	11	1	0	0
CHEEK	JAW	25	11	1	0	0
EYE	FACE	29	11	1	0	0
BUTTOCKS	HIP	24	9	1	0	0
ELBOW	KNEE	19	9	0	1	1
BACK	SHOULDER	8	8	1	0	0
FOOT	TOE	8	8	1	0	0
ARM	SHOULDER	8	6	1	0	0
ARM	WRIST	7	6	1	0	0
HAND	WRIST	8	6	1	0	0
HEEL	ANKLE	11	6	1	1	0
MOUTH	TOOTH	9	6	1	0	0
ELBOW	ARM	5	5	1	0	0
ELBOW	WRIST	7	5	0	1	1
FACE	MOUTH	11	5	1	0	0
FOOT	HEEL	7	4	1	0	0
CHEEK	CHIN	6	3	1	0	0
ELBOW	HAND	4	3	0	0	0
FACE	JAW	3	3	1	0	0
FINGER	ARM	4	3	0	0	0
FOOT	ANKLE	3	3	1	0	0
HEEL	LEG	3	3	1	0	0
KNEE	ANKLE	3	3	0	1	1
BACK	BELLY	1	2	1	0	0
BACK	BUTTOCKS	2	2	1	0	0
BACK	FOOT	2	2	0	1	0
BACK	HIP	2	2	1	0	0
CHEST	NIPPLE	2	2	1	0	0
ELBOW	NECK	2	2	0	0	0
FOOT	KNEE	2	2	0	0	0
HEAD	FOREHEAD	2	2	1	0	0
HEAD	TOOTH	2	2	1	0	0
HIP	LEG	3	2	1	0	0
KNEE	HEEL	2	2	0	1	0
KNEE	LEG	2	2	1	0	0
LEG	TOE	2	2	0	0	0
MOUTH	JAW	4	2	1	0	0
MOUTH	TONGUE	4	2	1	0	0
ARM	HIP	1	1	0	0	0
ARM	LEG	1	1	0	1	0
BACK	ARM	1	1	0	0	0
BACK	HAND	1	1	0	0	0
BACK	KNEE	1	1	0	0	0
BACK	LEG	1	1	1	0	0
BACK	MOUTH	1	1	0	0	0
BELLY	BUTTOCKS	1	1	0	1	0
BELLY	ELBOW	1	1	0	0	0
BELLY	NAVEL	1	1	1	0	0

BELLY	NECK	1	1	0	0	0
BUTTOCKS	HEEL	1	1	0	1	0
BUTTOCKS	LEG	1	1	1	0	0
CHEEK	BUTTOCKS	1	1	0	1	0
CHEEK	HAND	1	1	0	0	0
CHEEK	LIP	1	1	1	0	0
CHEEK	NAVEL	1	1	0	0	0
CHEST	SHOULDER	1	1	1	0	0
CHIN	LIP	2	1	1	0	0
CHIN	MOUTH	2	1	1	0	0
CHIN	TOOTH	1	1	1	0	0
EAR	EYE	1	1	1	0	0
EAR	NECK	1	1	1	0	0
ELBOW	ANKLE	1	1	0	1	1
ELBOW	BUTTOCKS	1	1	0	0	0
ELBOW	EYE	1	1	0	0	0
ELBOW	FINGER	1	1	0	0	0
ELBOW	HEAD	1	1	0	1	0
ELBOW	HEEL	4	1	0	1	0
ELBOW	SHOULDER	1	1	0	1	1
EYE	BREAST	1	1	0	0	0
EYE	EYELASH	1	1	1	0	0
EYE	FOOT	1	1	0	0	0
EYE	HEAD	1	1	1	0	0
EYE	KNEE	1	1	0	0	0
EYE	NIPPLE	1	1	0	1	0
FACE	LIP	1	1	1	0	0
FACE	NIPPLE	1	1	0	0	0
FACE	NOSE	1	1	1	0	0
FINGER	FOOT	1	1	0	0	0
FINGER	WRIST	1	1	1	0	0
FOOT	HAND	1	1	0	0	1
HAND	HIP	1	1	0	0	0
HAND	LEG	1	1	0	0	0
HAND	TOE	1	1	0	0	0
HEAD	BREAST	1	1	0	1	0
HEAD	KNEE	1	1	0	1	0
HEAD	NOSE	1	1	1	0	0
HEEL	SHOULDER	1	1	0	1	0
HIP	ANKLE	1	1	0	0	1
JAW	LIP	2	1	1	0	0
LEG	ANKLE	1	1	1	0	0
MOUTH	NECK	1	1	1	0	0
NECK	JAW	1	1	1	0	0
NECK	LEG	3	1	0	1	0
NECK	SHOULDER	1	1	1	0	0
NECK	TOE	1	1	0	0	0
NOSE	FOREHEAD	2	1	1	0	0
NOSE	TOOTH	1	1	0	1	0
TONGUE	LEG	1	1	0	0	0
TONGUE	NIPPLE	3	1	0	0	0
TOOTH	BREAST	2	1	0	0	0

References

1. Mayer, T., List, J.-M., Terhalle, A. & Urban, M. An Interactive Visualization of Crosslinguistic Colexification Patterns. In Hautli-Janisz, A., Lyding, V. & Rohrdantz, C. (eds.) *Proceedings of the LREC Workshop 'VisLR: Visualization as Added Value in the Development, Use and Evaluation of Language Resources'*, 1–8 (European Language Resources Association, Reykjavik, Iceland, 2014).
2. List, J.-M. Towards a History of Concept List Compilation in Historical Linguistics. *Hist. Philos. Lang. Sci.* (2018).
3. Rzymski, C. *et al.* The Database of Cross-Linguistic Colexifications, Reproducible Analysis of Cross-Linguistic Polysemies. *Sci. Data* **7**, 1–12, DOI: 10.1038/s41597-019-0341-x (2020).
4. Rosvall, M. & Bergstrom, C. T. Maps of Random Walks on Complex Networks Reveal Community Structure. *Proc. Natl. Acad. Sci.* **105**, 1118–1123, DOI: 10.1073/pnas.0706851105 (2008).
5. Newman, M. *Networks: An Introduction* (Oxford University Press, Oxford, 2018-[2010]), 2nd edition edn.
6. Jackson, J. C. *et al.* Emotion Semantics Show Both Cultural Variation and Universal Structure. *Science* **366**, 1517–1522, DOI: 10.1126/science.aaw8160 (2019).

7. List, J.-M., Greenhill, S. J. & Gray, R. D. The potential of automatic word comparison for historical linguistics. *PLOS ONE* **12**, 1–18 (2017).
8. Csárdi, G. & Nepusz, T. The igraph Software Package for Complex Network Research. *InterJournal Complex Syst.* **1695**, DOI: 10.5281/zenodo.3630268 (2006).
9. List, M. Sequence Comparison in Historical Linguistics (2014).
10. Romano, S., Vinh, N. X., Bailey, J. & Verspoor, K. Adjusting for Chance Clustering Comparison Measures. *J. Mach. Learn. Res.* **17**, 1–32 (2016).
11. Bodt, T. A. New Kusunda Data: A List of 250 Concepts. *Comput. Lang. Comp. Pract.* **3**, 1–4 (2020).
12. Abraham, B., Kinny, E., Zeliang, I. & Sako, K. *Sociolinguistic Research Among Selected Groups in Western Arunachal Pradesh: Highlighting Monpa* (SIL International, Dallas, 2018).
13. Allen, B. *Bai Dialect Survey* (SIL International, Dallas, 2007).
14. Greenhill, S. J. & Gray, R. D. *Bantu Basic Vocabulary Database* (Max Planck Institute for Evolutionary Anthropology, Leipzig, 2015).
15. Běijı̄ng Dàxué, U. o. B. (ed.) *Hànyǔ Fāngyán Cíhuì [Chinese Dialect Vocabularies]* (Wenzi Gaige, Beijing, 1964).
16. Bodt, T. A. & List, J.-M. Testing the Predictive Strength of the Comparative Method: An Ongoing Experiment on Unattested Words in Western Kho-Bwa Languages. *Pap. Hist. Phonol.* **4**, 22–44, DOI: 10.2218/pihph.4.2019.3037 (2019).
17. Bowern, C. & Atkinson, Q. Computational Phylogenetics and the Internal Structure of Pama-Nyungan. *Language* **88**, 817–845, DOI: 10.1353/lan.2012.0081 (2012).
18. Castro, A. & Pan, X. *Sui Dialect Research* (SIL International, Dallas, 2015).
19. Castro, A., Crook, B. & Flaming, R. *A Sociolinguistic Survey of Kua-Nsi and Related Yi Varieties in Heqing County, Yunnan Province, China* (SIL International, Dallas, 2009).
20. Castro, A. & Hansen, B. *Hongshui He Zhuang Dialect Intelligibility Survey* (SIL International, Dallas, 2010).
21. Chén, Q. *Miáoyáo Yǔwén [Miao and Yao Language]* (Zhōngyāng Mínzú Dàxué [China Minzu University Press], Beijing, 2012).
22. Language and Social Development Organization. *Chin Dialect Data Collection* (Language and Social Development Organization, Yangon, 2019).
23. Clark, E. A Phonological Analysis and Comparison of Two Kim Mun Varieties in Laos and Vietnam (2008).
24. Hale, A. *Clause, Sentence, and Discourse Patterns in Selected Languages of Nepal. Part IV. Wordlists*. SIL International Publications in Linguistics (Summer Institute of Linguistics of the University of Oklahoma, Norman, 1973).
25. Hantgan, A. & List, J.-M. Bangime: Secret Language, Language Isolate, or Language Island? A Computer-Assisted Case Study. *Pap. Hist. Phonol.* **7**, 1–43, DOI: 10.2218/pihph.7.2022.7328 (2022).
26. Huber, R. Q. & Reed, R. B. *Vocabulario Comparativo: Palabras Selectas de Lenguas Indígenas de Colombia [Comparative Vocabulary: Selected Words from the Indigeneous Languages of Columbia]* (Asociátion Instituto Lingüístico de Verano, Santafé de Bogota, 1992).
27. Hsiu, A. The Classification of Na Meo, a Hmong-Mien Language of Vietnam. In *25th Annual Meeting of the Southeast Asian Linguistics Society (SEALS 25)*, DOI: 10.5281/zenodo.1127804 (Payap University, Chiang Mai, Thailand, 2015).
28. Bowern, C., Epps, P., Hill, J. & Hunley, K. *Languages of Hunter-Gatherers and Their Neighbors: A Collection of Lexical, Grammatical, and Other Information About Languages Spoken by Hunter-Gatherers and Their Neighbors* (The University of Texas at Austin, Austin, 2021).
29. Johansson, N. E., Anikin, A., Carling, G. & Holmer, A. The Typology of Sound Symbolism: Defining Macro-Concepts Via Their Semantic and Phonetic Features. *Linguist. Typology* **24**, 253–310, DOI: 10.1515/lingty-2020-2034 (2020).
30. Kraft, C. H. *Chadic Wordlists* (Dietrich Reimer, Berlin, 1981).
31. Lindsey, K. L. Ende (Papua New Guinea). In Key, M. R. & Comrie, B. (eds.) *The Intercontinental Dictionary Series*, DOI: 10.5281/zenodo.4280602 (Max Planck Institute for Evolutionary Anthropology, Leipzig, 2021).
32. List, J.-M. Investigating the Impact of Sample Size on Cognate Detection. *J. Lang. Relatsh.* **11**, 91–102, DOI: 10.31826/jlr-2014-110111 (2014).
33. Luangthongkum, T. A View on Proto-Karen Phonology and Lexicon. *J. Southeast Asian Linguist. Soc.* **12**, i–lii (2019).
34. Mann, N. W. A Phonological Reconstruction of Proto Northern Burmic (1998).
35. Marrison, G. E. *The Classification of the Naga Languages of North-East India* (School of African and Oriental Sciences, London, 1967).
36. Mitterhofer, B. *Lessons from a Dialect Survey of Bena: Analyzing Wordlists* (SIL International, Dallas, 2013).
37. Nagano, Y. & Prins, M. *rGyalrongic Languages Database* (National Museum of Ethnology, Osaka, 2013).
38. Dellert, J. *et al.* NorthEuraLex: A Wide-Coverage Lexical Database of Northern Eurasia. *Lang. Resour. Eval.* **54**, 273–301, DOI: 10.1007/s10579-019-09480-6 (2020).
39. Koelle, S. W. *Polyglotta Africana or Comparative Vocabulary of Nearly Three Hundred Words and Phrases in More Than One Hundred Distinct African Languages* (Church Missionary House, London, 1854).
40. Robinson, L. C. & Holton, G. Internal Classification of the Alor-Pantar Language Family Using Computational Methods Applied to the Lexicon. *Lang. Dyn. Chang.* **2**, 123–149, DOI: 10.1163/22105832-20120201 (2012).
41. Sagart, L. *et al.* Dated Language Phylogenies Shed Light on the Ancestry of Sino-Tibetan. *Proc. Natl. Acad. Sci. United States Am.* **116**, 10317–10322, DOI: 10.1073/pnas.1817972116 (2019).
42. Sims, N. A. Reconsidering the Diachrony of Tone in Rma. *J. Southeast Asian Linguist. Soc.* **13**, 53–85 (2020).
43. So-Hartmann, H. Notes on the Southern Chin Languages. *Linguist. Tibet. Area* **11**, 98–119 (1988).
44. Sūn, H. *Zangmianyu Yuyin He Cihui [Tibeto-Burman Phonology and Lexicon]* (Chinese Social Sciences Press, Beijing, 1991).

45. Nurse, D. & Philippson, G. *The Tanzanian Language Survey* (Department of Foreign Languages and Linguistics of the University of Dar es Salaam, Dar es Salaam, 1975).

46. Greenhill, S. J. TransNewGuinea.org: An Online Database of New Guinea Languages. *PLOS ONE* **10**, 1–17, DOI: 10.1371/journal.pone.0141563 (2015).

47. Gerardi, F., Reichert, S. & Coelho Aragon, C. TuLeD (Tupían Lexical Database): Introducing a Database of a South American Language Family. *Lang. Resour. Eval.* **55**, 1–19 (2021).

48. Oskolskaya, S., Koile, E. & Robbeets, M. A Bayesian Approach to the Classification of Tungusic Languages. *Diachronica* **39**, 128–158, DOI: 10.1075/dia.20010.osk (2022).

49. Visser, E. Kalamang. In Key, M. R. & Comrie, B. (eds.) *The Intercontinental Dictionary Series*, DOI: 10.5281/zenodo.4518506 (Max Planck Institute for Evolutionary Anthropology, Leipzig, 2021).

50. Wang, F. Language Contact and Language Comparison. The Case of Bai (2004).

51. Haspelmath, M. & Tadmor, U. (eds.) *Loanwords in the World's Languages* (De Gruyter Mouton, Berlin, 2009).

52. Yang, C. Lalo Regional Varieties: Phylogeny, Dialectometry and Sociolinguistics (2011).

53. Yang, C. The Phonetic Tone Change *high > rising: Evidence from the Ngwi Dialect Laboratory. *Diachronica* **39**, 226–267, DOI: 10.1075/dia.19062.yan (2022).

54. Yu, H.-j. & Wang, Y. Mandarin Chinese. In Key, M. R. & Comrie, B. (eds.) *The Intercontinental Dictionary Series*, DOI: 10.5281/zenodo.4570270 (Max Planck Institute for Evolutionary Anthropology, Leipzig, 2021).

55. Z'graggen, J. A. *A Comparative Word List of the Rai Coast Languages, Madang Province, Papua New Guinea* (Australian National University, Canberra, 1980).

56. Othaniel, N. K. A Phonological Comparative Study of the Jen Language Cluster (2017).

57. Lama, Z. Q.-F. Subgrouping of Nisoic (Yi) Languages: A Study from the Perspectiv Eof Shared Innovation and Phylogenetic Estimation (2012).

58. Savelyev, A. & Robbeets, M. Bayesian Phylolinguistics Infers the Internal Structure and the Time-Depth of the Turkic Language Family. *J. Lang. Evol.* **5**, 39–53, DOI: 10.1093/jole/lzz010 (2020).

59. Key, M. R. & Comrie, B. *The Intercontinental Dictionary Series (Version 4.1)* (Max Planck Institute for Evolutionary Anthropology, Leipzig, 2015).

60. Zhivlov, M. *Annotated Swadesh Wordlists for the Ob-Ugrian Group (Uralic Family)* (The Global Lexicostatistical Database, Moscow, 2011).

61. Zhou, Y. Proto-Bizic. a Study of Tujia Historical Phonology (2020).

62. List, J.-M. *et al.* *Concepticon. A Resource for the Linking of Concept Lists (Version 2.5.0)* (Max Planck Institute for Evolutionary Anthropology, Leipzig, 2021).
